# Editorial Brevities

**Published:** 1887-10

**Authors:** 


### EDITORIALS AND MISCELLANEOUS.

### EDITORIAL BREVITIES.

MISTAKE.—The introductory lecture and opening exercises of the Southern Medical College will take place on the 4th of October, instead of the 5th, as stated in our last.

THE PIEDMONT FAIR.—We hope to meet many of our friends and subscribers at the approaching Fair. Call at our room, 75 South Pryor, and let us shake your hand.

JACOBS' PHARMACY COMPANY.—We invite special attention to the above enterprising house, on corner of Marietta and Peachtree streets, Atlanta. They are active, polite and accommodating gentlemen, very attentive to business, and their prices are very moderate.

MEDICAL AND MISCELLANEOUS BOOKS.—Read carefully the advertisement on page 26 of Messrs. Lester & Kuhrt, No. 7 Whitehall street. These gentlemen are courteous and attentive to purchasers, and keep a large supply of books of all kinds. Students can here procure medical books at the lowest cash prices.

DEATH OF DR. BORCHEIM.—We regret to have to report the death, by suicide, of Dr. Borcheim, of Atlanta, on September 11, 1887.

He was an intelligent practitioner and a worthy member of the profession, but had unfortunately contracted the cocaine habit, to which is attributed that morbid mental condition which led to the suicide.

DR. O. H. TALIAFERRO, an eminent physician of Atlanta, and Professor of Obsteterics in the Atlanta Medical College, died recently at Tate Springs, Tennessee.

Dr. Taliaferro was a man of prominence in the profession, and had acquired quite a distinguished reputation, especially in the department of gynæcology. As a lecturer he stood well and will be sorely missed in the Atlanta Medical College and by numerous friends and patrons in Atlanta and throughout the South. He was truly a good man. Peace to his memory.

DEATH OF DR. JAMES A. GRAY.—Just as we go to press we are informed of another sad dispensation in the medical profession of our city, the death of Dr. James A. Gray. a prominent physician, one of the editors of the *Atlanta Medical Journal*, and Secretary of the Georgia Medical Association. He died of typhoid fever at his residence in this city on the morning of September 27, 1887. He leaves an affectionate wife and many relatives and numerous friends to mourn his departure.

PHOTO OF THE DOCTORS.—We are pleased to acknowledge the receipt of a photo showing the members of the Georgia Medical Association present at the second day of the Association, at the meeting in Atlanta, in April, 1887. For this kindness we are indebted to the excellent house of Parke, Davis & Co., of Detroit, and sent us by their gentlemanly agent, Dr. J. W. Stone, of Atlanta. We shall cherish it as a pleasing courtesy from a high-toned, liberal business firm, which has done and is doing much to advance the interest of pharmacy and the medical profession, and as a souvenir of a most delightful entertainment, given to the Georgia Medical Association, by E. W. Marsh & Co., proprietors of the celebrated Salt Springs.

A NOVEL DEPARTURE IN ADVERTISING.—Parke, Davis & Co. propose to inaugurate rather a novel departure in advertising. It is their intention to publish in the advertising pages they occupy in medical journals a series of what they term plain talks to physicians—in each issue taking up a certain class of preparations and pointing out the reasons why they deserve to be prescribed, until all their preparations shall have thus been presented. The excellence of the products of this house are well known and it is to be presumed that their long experience in the manufacture of medicines will enable them to say in these informal talks something of real interest and benefit to their medical friends.

